# *PLOS Neglected Tropical Diseases* 2016 Reviewer and Editorial Board Thank You

**DOI:** 10.1371/journal.pntd.0005469

**Published:** 2017-03-20

PLOS and the *PLOS Neglected Tropical Diseases* (*PLOS NTDs*) team would like to express our sincere gratitude to everyone who lent us their expertise as an editor, as a guest editor, or as a peer reviewer in 2016. We are fully dependent on the voluntary efforts of these experts to publish the journal for the academic community. Over the past year, *PLOS NTDs* relied on 405 editorial board members and guest editors to manage the 1,771 articles submitted to the journal, which were assessed by more than 2,500 reviewers. Their efforts enabled the publication of more than 870 rigorous, Open Access papers.

The names of our 2016 editors that handled submitted manuscripts appear in the Supporting Information as S1 Editor List and as S1 Guest Editor List. Our reviewers appear in the Supporting Information as S1 Reviewer List. We are grateful for their dedication, generosity, and support of Open Access science. Thank you all.

## Supporting information

S1 Editor List Alphabetical list of 2016 *PLOS NTDs* Editorial Board Members. (PDF)

S1 Guest Editor List Alphabetical list of 2016 *PLOS NTDs* Guest Editors. (PDF)

S1 Reviewer List Alphabetical list of 2016 *PLOS NTDs* peer reviewers. (PDF)
