# Supplementary material for: PLOS Neglected Tropical Diseases 2016 Reviewer and Editorial Board Thank You
Source: PLoS Negl Trop Dis. 2017 Mar 20;11(3):e0005469. doi: 10.1371/journal.pntd.0005469 (PMC5358734; doi:10.1371/journal.pntd.0005469)
Supplement: S1 Guest Editor List — (PDF) [file pntd.0005469.s002.pdf]

PLOS | NEGLECTED TROPICAL DISEASES

PLOS Neglected Tropical Diseases would like to thank all those who served as Guest Associate Editors in 2016:

Adly Abd-Alla
David Addiss
Marco Albonico
Waleed Al-Salem
Charles Apperson
Philip Armstrong
Stephen Attwood
Guillermina Avila
Kelly Baker
Claudio Bandi
Gad Baneth
Alan Barrett
Dirk Berkvens
Stuart Blacksell
Moses Bockarie
Mariangela Bonizzoni
Christian Bottomley
Amaya Bustinduy
Conor Caffrey
Melissa Caimano
Juan Calvete
Yves Carlier
Jane Carlton
Vern Carruthers
Adriano Casulli
Arturo Centurion-Lara
Jong-Yil Chai
Eric Chatelain
Mitali Chatterjee
Donato Cioli
Sarah Cleaveland
Julie Clennon
Joachim Clos
Albert Descoteaux
George Dimopoulos
Sheila Donnelly
Sandy Douglas
Jennifer Downs
Gregory Ebel
Andrew Fenton
Marcelo Ferreira
Peter Fischer
Agnes Fleury
Jennifer Friedman
Hans-Peter Fuehrer
Michael Gaunt
Patrick Gérardin
Luigi Gradoni
Robert Greenberg
Margaret Gyapong
Asrat Hailu
Jo Halliday
Kate Halton
Mary Hayden
Celia Holland
Peter Horby
Olaf Horstick
Marc Hübner
Shannon Johnson
Emmitt Jolly
Thomas Junghanss
William Klimstra
Amy Klion
Alain Kohl
Sandhya Kortagere
Ulrich Kuch
Juan Laclette
Poppy Lamberton
Edmundo Larrieu
Laura Layland
Louis Maes
Mathieu Maheu-Giroux
Pablo Maravilla
Christina Marra
Aaron Maule
Henry McSorley
Rojelio Mejia
Edward Mitre
Jason Mulvenna
Peter Myler
Hira Nakhasi
Bryson Ndenga
Juan Olano
Chiquita Palha De Sousa
Anna Papa
William Petri Jr.
Maia Rabaa
Vedantam Rajshekhar
Ramesh Ratnappan
Sergio Recuenco
Robert Reiner
Gabriel Rinaldi
Syamal Roy
John Samuelson
Ana Sanchez
Manuel Schibler

Donald Shepard
Pei-Yong Shi
Cameron Simmons
Wilma Stolk
Yasuhiro Suzuki
Herbert Tanowitz
Paul Torgerson
Rebecca Traub
Moriya Tsuji
Hugo Turner
Lance Turtle
Johan van Griensven
Nikos Vasilakis
Susana Vaz Nery

Dominique Vuitton
Samuel Wanji
Brian Weiss
Guilherme Werneck
A. White Jr.
Thomas Wilke
Bridget Wills
Mark Wilson
Shona Wilson
Andrea Winkler
Elizabeth Winzeler
Laila Woc Colburn
Zhijie Zhang
Joseph Zunt
