# Supplementary material for: PLOS Neglected Tropical Diseases 2016 Reviewer and Editorial Board Thank You
Source: PLoS Negl Trop Dis. 2017 Mar 20;11(3):e0005469. doi: 10.1371/journal.pntd.0005469 (PMC5358734; doi:10.1371/journal.pntd.0005469)
Supplement: S1 Reviewer List — (PDF) [file pntd.0005469.s003.pdf]

PLOS Neglected Tropical Diseases would like to thank all those who reviewed on behalf of the journal in 2016:

Kjersti Aagaard
John Aaskov
Xavier Abad
Elfadil Abass
Ibrahim Abassi
Emmanuel Abatih
Mebrahtu Abay
Aymn Abbas
Syed Abbas
Mariamo Abdala Mbofana
Khaled A. Abdel-Moein
Azad Abdu
Eniola Abe
Bernadette Abela-Ridder
Judith Aberle
Jason Abernathy
Ajibola Abioye
David Abraham
Candida Abreu
Patrícia Abreu
Nicole Achee
Rodney Adam
John Adams
Linda Adams
Vanessa Adaui Sicheri
Joel Addawe
David Addiss
Ayola Adegnika
Coen Adema
Daniel Adesse
Babatunde Adewale
Isaac Adewale
Tomabu Adjobimey
Philip Adongo
Vincent Adung'a
Luis Afonso
Suneth Agampodi
Eric Agola
Javed Agrewala
Omar Ahmad
Rushdy Ahmad
Ahmed Ahmed
Be-Nazir Ahmed
Kamruddin Ahmed
Lin Ai
Steven Aird
Sitara Ajjampur
Okan Akhan
Amir Ahmad Akhavan
Pierre Akilimali
Ramesh Akkina
Rama Akondy
Francis Akor
Serap Aksoy
Mohammad Alam
Al-Sayed R. Al-Attar
Francisco Alcalde
Javier Alda
Jerry Aldridge
Jared Aldstadt
Sami Al-Dubai
James Alexander
Neal Alexander
Jorge Alfaro-Murillo
Kendra Alfson
Jackeline Alger
Andy Alhassan
Asad Ali
Nahid Ali
Matthew Aliota
Emilie Alirol
Fiona Allan
Orchid Allicock
Salvador Almagro-Moreno
Igor Almeida
Rodrigo Almeida-Paes
Hesham Al-Mekhlafi
David Alonso
Guillermo Alonso
Julio Alonso-Padilla
Luke Alphey
Khaled M. Al-Qaoud
Sam Alsford
Christian Althaus
Daniel Altmann
Barry Alto
José M. Alunda
Julio Alvarez
Vanina Alvarez
Cristian Alvarez Rojas

Fernando Alvarez-Valin
Fabiana Alves
Marcos Amaku
Ananda Amarasinghe
Ritu Amatya
Kei Amemiya
Nana Ama Amissah
Yaw Amoako
Francis Amrit
John Amuasi
Mary Amuyunzu-Nyamongo
John Andersen
Burt Anderson
Deborah Anderson
John Anderson
Kathryn Anderson
Shuji Ando
Ted Andreadis
Elizabeth Andrews
Jason Andrews
Andrea Angheben
Rashid Ansumana
Karim Aoun
Juan Aparicio
Ruslan Aphasizhev
Charles Apperson
Victor Aquino
Raouf Arafat
Byron Arana
Vidya Arankalle
Bruno Arca
Ana Paula Arez
Ariaranee Ariaratnam
Kevin Ariën
Amit Arjyal
Peter Armbruster
Nicole Arrigo
Carmen Arriola
Sassan Asgari
Rana Jawad Asghar
Anthony Ashton
Emmanuel Assampong
Geoffrey Attardo
Alexandra Aubry
Sarah Auburn
Furaha August
Albert Auguste
Vicky Avery
Guillermina Avila
Ali Ayadi
C.F.J. Ayres
Abdu Azad
Simon Babayan
Subash Babu
Olivia Bacellar
Eric Bachelder
Roberto Badaró
Young Mee Bae
Alan Baer
Karoun Bagamian
J. Baird
Kelly Baker
Margaret Baker
Tamas Bakonyi
Aliyu Bala
Mangai Balasegaram
Jonathan Ball
Guy Ballard
Gad Baneth
Ashley Banyard
Bernardo Baradat Gonzalez
Clara Barbiéri
Angela Barbosa
David Barbosa
Dulce Barbosa
Thales Barçante
Beatrice Barda
Carolina Barillas-Mury
Stephen Barker
Christian Barnabé
Yves Barogui
Josafá Barreto
Flavia Barreto Dos Santos
Alan Barrett
Alejandra Barrio
Lyric Bartholomay
Christopher Basler
Patrick Bastien
Leonardo Bastos
Anirban Basu
Carolina Batista
Dennis Baumgardner
Andreas Baumler
Iliana Baums
Daniel Bausch
Sina Bavari
Christopher Bayne
Mathew Beale
Frank Beard
Andrea Beaton
Pauline Beattie
P. Beatty

Coreen Beaumier
Ingeborg Becker
Charmagne Beckett
Robin Beech
Mike Begon
Daniel Beiting
Vivian Bellofatto
Silvana Belo
Slimane Ben Miled
Afif Ben Salah
D. Benacer
Gil Benard
Mark Benbow
Scott Bender
Sarah Bennett
Sasisekhar Bennuru
Shalom Ben-Shimol
Dennis Bente
Mary Berbee
Caryn Bern
Scott Bernhardt
Paul Bessell
Sonja Best
Bernard Bett
Judy Bettridge
Stephen Beverley
Tariku Beyene
Parna Bhattacharya
Tapan Bhattacharyya
Helle Bielefeldt-Ohmann
Beverley-Ann Biggs
Peter Billingsley
Marco Binder
Nana-Kwadwo Biritwum
Kimberly Bishop-Lilly
Zeno Bisoffi
Sylvie Bisser
William Black IV
Stuart Blacksell
Carol Blair
Alexandre Blake
Damer Blake
Isobel Blake
Daniel Blanchard
David Bland
David Blaney
Lucas Blanton
José-María Blasco
Mark Blaxter
David Blazes
Thomas Bleck
Ian Blenkharn
Joachim Blocher
David Blok
Marshall Bloom
Brittany Blouin
Johannes Blum
Lucille Blumberg
Daniel Boakye
Cristina Bocanegra
Pierre-Yves Bochud
Pierre-Yves Boelle
Christian Bogdan
Andrea Boggild
Jonathan Bohbot
Erwin Bohn
Sophie Boisson
Wagner Bonat
A. Bonifaz
Mariangela Bonizzoni
Thidarut Boonmars
Rebecca Borchering
Seth Bordenstein
Valeria Borges
Piotr Borkowski
Irene Bosch
Monica Botelho
Emmanuel Bottieau
Carlos Botto
Christian Bottomley
Olivier Bouchaud
Sonia Boughattas
Henri-Jean Boulouis
Aida Bouratbine
Hervé Bourhy
Pascale Bourhy
Kostas Bourtzis
Teun Bousema
Michel Boussinesq
Amina Boutellis
Jérémy Bouyer
Saeid Bouzari
Richard Bowen
William Bower
Dwight Bowman
Leigh Bowman
Ross Boyce
Kenneth Boyer
Rosemary Boyton
Zbynek Bozdech
Marcelo Bozza
Doug Brackney

Richard Bradbury
Mark Bradley
Molly Brady
Oliver Brady
Érika Braga
Ekanem Braide
Paulo Brandao
Wim Brandsma
Sara Brant
Aaron Brault
Reginaldo Brazil
Benjamin Brennan
Patrick Brennan
Matthew Brewer
William Brieger
Olivier Briët
Deborah Briggs
Paul Brindley
Robert Brinkerhoff
Seth Britch
Constança Britto
Collette Britton
Mathieu Brochet
Christopher Broder
Cláudia Brodskyn
Simon Brooker
Peter Brophy
Catherine Brown
Grant Brown
Eric Brum
Alejandro Brun
Reto Brun
Enrico Brunetti
Robert Brunham
Bruno Bucheton
Amy Buck
Frederick Buckner
Philip Budge
Pierre Buekens
Lilian Bueno
Michael Buhnerkempe
Salome Bukachi
Michael Bukrinsky
John Buntine
Tristan Burgess
Austin Burt
Sakib Burza
Anne Buschmann
Amanda Bustinduy St. Georges
James Butler
Tom Butler
Ghazwan Butrous
Alison Buttenheim
Miguel Cabada
Conor Caffrey
Guy Caljon
Amanda Calvert
Juan Calvete
Manuel Calvopina
Mattia Calzolari
Vitaliano Cama
Anton Camacho
Luiz Camacho
Caroline Cameron
Ewan Cameron
Mary Cameron
Cyril Caminade
Corey Campbell
Lindsay Campbell
Suzy Campbell
Raul Campos
Anthony Cannella
Luz Cano
Paul Cantey
Jian-Ping Cao
Rosario Capeding
Michael Cappello
Beniamino Caputo
Mabel Carabali
Hélène Carabin
Eric Caragata
Marta Cardinal
Nora Cardona-Castro
Luis Cardoso
James Carey
Cristina Carias
Yves Carlier
Jason Carlyon
David Carmena
Elisabeth Carniel
Giovanna Carpi
Arturo Carpio
Wendy Carr
Hernán Carrasco
Vanessa Carregaro
Eugenia Carrillo
Lauren Carrington
Mark Carrington
Dee Carter
K.C. Carter
Teresa Carvalho
Lisa Casanova

Edoardo Casiglia
Lauren Castro
Adriano Casulli
Isabella Cattadori
Simon Cauchemez
Lisa Cavacini
Colleen Cebulla
Giuliano Cecchi
Leticia Cedillo-Barron
Arturo Centurion-Lara
Laura Cervi
Dave Chadee
Ann Chahroudi
Jong-Yil Chai
Rana Chakrabarti
Jean Challacombe
Clara Champagne
Ta-Chien Chan
Aileen Chang
Gwong-Jen Chang
Kwang-Poo Chang
Narisara Chantratita
Dennis Chao
François Chappuis
Eric Chatelain
Mitali Chatterjee
Soumya Chatterjee
Gautam Chaudhuri
Ana Thereza Chaves
Luis Fernando Chaves
Ian Cheeseman
Rubing Chen
Yee-Chun Chen
Erika Chenais
Allen Cheng
Qin Cheng
Qu Cheng
Pascal Cherpillod
Cédric Chesnais
Francisco Chiaravalloti Neto
R. Matthew Chico
Wirongrong Chierakul
James E. Childs
T. Chiller
Sylvester Chima
Wei-Mei Ching
Faraimunashe Chirove
Chetan Chitnis
Nakul Chitnis
Miguel Chiurillo
Nam-Hyuk Cho
Sang-Nae Cho
Bruno Chomel
Rajib Chowdhury
Gerardo Chowell
Henry Choy
Garret Christensen
Rebecca Christofferson
Hung-Yi Chuang
Joseph Chuckwu
Iza Ciglenecki
Alexander Ciota
Daniela Cirillo
Guéladio Cissé
Cornelius Clancy
Hannah Clapham
C. Graham Clark
Carolyn Clark
Gary Clark
Penny Clarke
Thomas Clasen
Sarah Cleaveland
Archie Clements
Jan Clerinx
Gilles Clermont
Joachim Clos
Joel Coats
Claudia Codeço
James Cody
Eduardo Coelho
Flavio Coelho
Luc Coffeng
Lark Coffey
Stewart Cole
Robert Colebunders
Daniel Colley
Matthew Collins
James Collins III
Felipe Colón-González
Tonya Colpitts
Diego Comerci
Marcelo Comini
Fátima Conceição-Silva
Andrew Conlan
Roxanne Connelly
Franz Conraths
Paul Converse
David Conway
Klaus Conzelmann
Alex Cook
Peter Cook
Ben Cooper

Phil Cooper
Marc Coosemans
Richard Cordaux
Betina Corsico
Paul Corstjens
Ileana Corvo
Paul Cos
Stephen Cose
Jean-Francois Cosson
Carlos Costa
Federico Costa
Nagilafrancinete Costasecundino
James Cotton
Jean Coulibaly
José Coura
Aurélie Courcoul
Bertrand Courtioux
Iliano Coutinho-Abreu
Chris Cowled
Siobhan Cowley
Christina Coyle
Philip Craig
Philip Crain
Mike Cranfield
Charles Criscione
Maria Cristiano
Simon Croft
Elizabeth Cromwell
Ian Crozier
Angela Cruz
Israel Cruz
Jorge Cruz-Reyes
Zulma Cucunuba
Yujun Cui
Erika Cule
Marcel Cunha
Edecio Cunha-Neto
Adam Cunningham
Jane Cunningham
Lucas Cunningham
Eddie Cupp
Jeffrey Currier
Val Curtis
Sally Cutler
Krystyna Cwiklinski
Alda Maria Dacruz
Johanna Daily
Pat Dale
Timothy Dallman
Stefano D'Amelio
David Dance
Hector Dantes
Wanderson DaRocha
Thomas Darton
Toni Darville
Jyoti Das
Murari Das
Pranab Kumar Das
Silvia Da-Silva
Virginia Dato
Regina Daumas
Miles Davenport
Gail Davey
Dan David
John David
Andrew Davidson
C. Todd Davis
Stephanie Davis
Katinka De Balogh
Zoilo de Camargo
Reginald De Deken
William de Glanville
Bouke de Jong
Valeria Marcal de Lima
Herbert de Matos Guedes
Thierry De Meeus
Diogo de Melo
Natalia de Miguel
Isabel de Miranda Santos
Geraldine De Muylder
Camila de Oliveira
Adolfo de Roodt
N.R. de Silva
Dziedzom de Souza
Fabiana Covolo de Souza-Santana
Annabelle de St. Maurice
Sophia de Vries
Deborah Dean
Natalie Dean
Amanda Debes
Anjan Debnath
George Deepe Jr.
Victor Defilippis
Abraham Degarege
Randall Dejong
Rosa Maria del Angel
Oscar Del Brutto
Luis del Carpio-Orantes
Alessandra della Torre
M. Delpino
Margriet Den Boer
Shuwen Deng

Arminder Deol
Xavier Deparis
Peter Deplazes
Albert Descoteaux
Philippe Desprès
Mahalia Desruisseaux
Eileen Devaney
Gregor Devine
Brecht Devleesschauwer
Benjamin Dewals
Ranadhir Dey
Varough Deyde
Roberto Di Santo
Diawo Diallo
Mawlouth Diallo
Michael Diamond
Alvaro Diaz
Antonino Dicaro
Katherine Dickinson
Sean Diehl
Bernhard Dietzschold
Rod Dillon
Nedialko Dimitrov
George Dimopoulos
Andrew Dinardo
Diwakar Dinesh
Qiliang Ding
Particio Diosque
Ermias Diro
Colette Dissous
Sabine Dittrich
Devika Dixit
Julianne Djordjevic
Félix Djossou
Yenny Djuardi
Rubens do Monte-Neto
Gauthier Dobigny
Deborah Dobson
Hazel Dockrell
Meritxell Donadeu
Maria Rita Donalisio
Sheila Donnelly
Jeffrey Donowitz
Pedro D'Orleans-Juste
Patricia Dorn
Pierre Dorny
Ellen Dotson
Sandy Douglas
Kimberly Dowd
Jennifer Downs
Philip Downs
Stephen Doyle
Lesley Drake
Christopher Drakeley
Michel Drancourt
Mark Drew
Gilles Dreyfuss
Ana Paula Duarte
Anuradha Dube
Jean-Francois Dubremetz
Jean Dubuisson
Patrick Duffy
J. Stephen Dumler
Eric Dumonteil
Susanna Dunachie
Ildiko Dunay
Veasna Duong
Sandra Durães
Anna Durbin
Salome Dürr
Jonathan Dushoff
Philippe Dussart
Michael Duszenko
Rebecca Dutch
Malcolm Duthie
Walderez Dutra
Joan Dzenowagis
Gregory Ebel
Bassey Ebenso
Mark Eberhard
Aurea Echevarria
Juan E. Echevarria
Philip Eckhoff
Michael Eddleston
Miriam Eddyani
Obaghe Edeghere
Melissa Edeling
Robert Edelman
Dileepa Ediriweera
Thomas Edwards
Michael Efunshile
Daniel Eibach
Christopher Eickhoff
Millicent Eidson
Lloyd Einsiedel
John Elder
Carole Eldin
Daniel Elias
Maria Carolina Elias
Carl-Gustaf Elinder
Simon Elliot
Alison Elliott

Esther Ellis
James Ellison
Sayda El-Safi
Najib El-Sayed
Kathryn Else
Paul Emerson
Aidan Emery
Samantha Emery
Matt Eng
Markus Engstler
Eva Enns
Mark Eppinger
Bobbie Erickson
Kacey Ernst
Hildegund Ertl
Juliana Estanislau
J. Esterhuizen
Darin Evans
Bart Everts
Sara Eyangoh
Amara Ezeamama
Luca Facchinelli
Helen Faddy
Ahmed Fahal
Keke Fairfax
Alan Fairlamb
Jessica Fairley
M. Abul Faiz
Franco Falcone
Joseph Falkinham
Padraic Fallon
Liqun Fang
Marcelo Fantappie
Ary Faraji
Nuno Faria
Andrew Farlow
Parissa Farnia
Maxwell Farrell
Carmen Faso
Guido Favia
Nicholas Feasey
David Fedson
David Fegan
Christine Fehlner-Gardiner
Leora Feldstein
Carl Feng
Florence Fenollar
Andrew Fenton
Fabio Fernandes
Christopher Fernandez Prada
Pedro Fernández-Soto
Lakkumar Fernando
Matthew Ferrari
Ana Ferreira
Joseli Ferreira
Marcelo Ferreira
Ricardo Ferreira
Sidney Ferreira
Lluis Ferrer
María Ferrer
Lori Ferrins
Mark Field
Matt Field
Joshua Fierer
Katherine Figarella
Luisa Figueiredo
Ulrike Fillinger
Katja Fink
Stefan Finke
Pier Luigi Fiori
Anne Fischer
Egil Fischer
Katja Fischer
Marc Fischer
Peter Fischer
William Fischer
Durland Fish
Matthew Fisher
Christopher Fitzpatrick
Colin Fitzsimmons
James Fleckenstein
Fiona Fleming
Courtney Fletcher
Tom Fletcher
Agnes Fleury
Laurence Flevaud
Ana Flisser
Eduardo Flores
Carlos A. Flores-Lopez
Monica Florin-Christensen
Robin Flynn
William Flynn
Dina Fonseca
Albin Fontaine
Krystal Fontaine
Luis Fonte
Anthony Fooks
Christen Fornadel
J. Forrest
Naomi Forrester
Brett Forshey
Anny Fortin

Jarrod Fortwendel
Norma Foss
Geraldine Foster
Pierre-Edouard Fournier
Rob Fowler
Kimberley Fox
LeAnne M. Fox
Spencer Fox
Brian Foy
Marco Frade
Gladis Fragoso
Genoveffa Franchini
Dimitrios Frangoulidis
Richard Franka
Alexander Franz
Malcolm Fraser
Matthew Freeman
Teresa Freire
Michael French
Manuel Fresno
Winifred Frick
David Friedman
Eleanor Friedman
Jennifer Friedman
Michael Frimpong
Sieghard Frischmann
Megan Fritz
Ilya Frolov
Elena Frolova
Roger Frutos
Linda Fryland
Zhen F. Fu
Samuel Fuhrimann
Shuetsu Fukushi
Claire Fuller
Isaac Chun-Hai Fung
João M Furtado
Janet Fyfe
José Gabrie
Sarah Gabriel
Albis-Francesco Gabrielli
Michaela Gack
Katya Galactionova
Montserrat Gállego
Robert Gallo
Renee Galloway
Francisco Gamarro
Yunn-Hwen Gan
Ekambaram Ganapathy
Sreenivas Gannavaram
Teresa Garate
Melissa Garcia
Manuel Garcia-Herranz
Loic Garçon
Robert Garry
Joaquim Gascon
Giuliano Gasperi
Katherine Gass
Salvacion Gatchalian
Derek Gatherer
Michelle Gatton
Michael Gaunt
Virendra Gautam
Philippe Gautret
Bruce Gaynor
Soraya Gaze
Pedro Gazzinelli-Guimarães
Alan Geater
Teshome Gebre
Stanny Geerts
Stefan Geiger
Brian Geiss
Peter Geldhof
Annemieke Geluk
Moti Yohannes Gemechu
Claudio Genchi
Nicholas Generous
Blaise Genton
Dylan George
Patrick Gérardin
Arthur Getis
Lorenzo Giacani
Evangelos Giamarellos-Bourboulis
Robert Gibbons
Sebastian Gibot
Andrew Gibson
Gabriella Gibson
Greg Gibson
Amy Gilbert
Nicolas Gilbert
Lars Gille
Joseph Gillespie
Robert Gilman
Geoffrey Gimonneau
Ricardo Giordano
Rodolfo Giunchetti
Pierre Gladieux
Gregory Glass
Kathryn Glass
Gerald Gleich
Dominik Glinz
Andrea Gloria-Soria

Anne Gnauck
Cyrille Goarant
Geoffrey Gobert
Ethan Goddard-Borger
Jacques Godfroid
Tony Goldberg
Ann Goldman
Delia Goletti
Kenneth Gollob
Bruno Gomes
João Gomes
Beatriz Gomez
Maria Adelaida Gomez
Ricardo Gomez
Maria Angeles Gómez-Morales
Ricardo Gonçalves
Cassiano Gonçalves-De-Albuquerque
Peng Gong
Nelder Gontijo
David González-Barrio
Enrique González-Tortuero
Rodion Gorchakov
Aubree Gordon
Marga Goris
Hiro Goto
Nicole Gottdenker
Eduardo Gotuzzo
Ernest Gould
Benjamin Gourbal
Sebastien Gourbiere
Brian Gowen
Emily Gower
Neena Goyal
Dennis Grab
Luigi Gradoni
Carlos Graeff-Teixeira
Loren Gragert
John Grainger
Marina Gramiccia
Francesco Grandesso
Warwick Grant
William Grant
Vyacheslav Grechenko
Robert Greenberg
Katie Greenland
Anthony Gregory
Frederic Grenouillet
Christoph Grevelding
Finn Grey
John Grieco
Paul Griffin
D. Griffith
Michael Grigg
Maria Eugenia Grillet
Felix Grimm
Tara Grinnage-Pulley
Gregory Gromowski
Aaron Gross
Uwe Gross
Jacques Grosset
Alessandra Guarneri
Jean-Francois Guegan
Vanina Guernier
Claudia Guezala
Felipe Guhl
Bruno Guigas
Javier Guitian
Keith Gull
Sharmini Gunawardena
Bhavna Gupta
David Gurarie
Margaret Gyapong
Lam Ha
Karen Haag
Abdulrazaq Habib
Esmael Habtamu
Danny Haddad
Andrew Haddow
Emily Hagan
Ferry Hagen
Asrat Hailu
Lee Haines
Ken Halanych
Yara Halasa
Andrew Hall
Rebecca Hall
Roy Hall
Jo Halliday
Katherine Halliday
Eric Halsey
Gabriel Hamer
Sarah Hamer
Katie Hampson
Penelope Hancock
Sukwan Handali
Kathryn Hanley
Chanditha Hapuarachchi
Rashidul Haque
John Hargrove
Gundel Harms
Billy Harnett
William Harnett

Jason Harris
Nicola Harris
Robert Harrison
Wiebke Hartmann
Hideki Hasegawa
Hassan Hashimi
Marisa Hast
Kenneth Hawkins
Roderick Hay
Mary Hayden
Daihai He
Julie Healer
Gerald Heckel
Miriam Heijnders
Mark Heise
Thiravat Hemachudha
Ryan Hemme
Andrew Hemphill
David Hendrickx
Cesar Henriquez-Camacho
Debroski Herbert
Julia Hermida
Mauricio Hernandez Avila
Socrates Herrera
Jesica Herrick
Jorg Heukelbach
James Hewitson
Robert Heyderman
Mallorie Hide
Rolf Hilgenfeld
Julian Hillyer
B. Joseph Hinnebusch
Thomas Hladish
Paulo Ho
Mary Hodges
Jane Hodgkinson
Martin Hoenigl
Heather Hoffman
Ary Hoffmann
Andreas Hofmann
Daniel Hoft
Robert Hogan
Cornelis Hokke
Michael Holbrook
Lindy Holden-Dye
Celia Holland
Martin Holland
Christoph Hölscher
Jennifer Honda
Yasushi Honda
Jared Honeycutt
Sung-Jong Hong
Sung-Tae Hong
Nildimar Honorio
Robert Hontz
Edward Hook
D. Craig Hooper
Adrian Hopkins
Martin Horn
William Horsnell
Olaf Horstick
John Horton
Eugenio Hottz
Genevieve Housman
Hao Hu
Wenbiao Hu
Jennifer Huang
Lu Huang
Wan-Ting Huang
Yan Huang
Marc Hübner
Karen Hughes
Martin Hugh-Jones
Leon Hugo
Stephane Hugonnet
Jean-Pierre Hugot
Raymond Hui
Elizabeth Hunsperger
Angela Huttner
Kris Huygen
Emily Hyle
Vaclav Hypsa
Atila Iamarino
Valerio Iebba
Eliane Ignotti
Tetsuro Ikegami
Timothy Inglis
Satoshi Inoue
Adamu Inuwa
Angela M Ionica
Sergey Iordanskiy
Deepak Ipe
Michael Irvine
Saiful Islam
Wannaporn Ittiprasert
Renata Ivanek
Ricardo Izurieta
Abdul Jabbar
Nabila Jabrane-Ferrat
Alan Jackson
Yves Jackson
Michael Jacobs

Robert Jacobs
Marc Jacobsen
Thomas Jaenisch
Joanne Jamie
Ana Jansen
Petrus Jansen Van Vuren
Maritza Jaramillo
Rick Jarman
Stefan Jaronski
Edward Jarroll
Aurelie Jeandron
Fakhri Jeddi
Stephen Jenkins
Selma Jeronimo
Ann Jerse
Nicholas Jewell
Ayan Jha
Ravi Jhaveri
Tie-Wu Jia
Lin-Hua Jiang
Shibo Jiang
M.D. Shariful Alam Jilani
Maria Isabel Jimenez
Veronica Jimenez Ortiz
Maria V. Johansen
Michael Johansson
Barbara W. Johnson
Karyn Johnson
Paul Johnson
Raymond Johnson
Douglas Jones
Kathryn Jones
Malcolm Jones
Heather Jordan
Luisa Jordao
Serene Joseph
Suresh Joshi
Peter Jourdan
Fernando Octávio Jucá Neto
Steven Juliano
Thomas Junghanss
Silvia Justi
Malika Kachani
Rebekah Kading
Masanori Kai
Christoph Kaiser
Laurent Kaiser
Maria Kaiser
Murali Kaja
Siripen Kalayanarooj
Mary Kamb
Joseph Kamgno
Taro Kamigaki
Ronald Kaminsky
Niranjan Kanesa-Thasan
Edna Kaneshiro
Congbao Kang
Seokyoung Kang
Flora Kano
Anu Kantele
Rowland Kao
Christian Kapel
James Kaper
Rinki Kapoor
Susanta Kar
Shahid Karim
Samuel Kariuki
Hiroaki Kariwa
Alexander Karlas
Anuradhani Kasturiratne
Joldoshbek Kasymbekov
Johanna Kattenberg
Leah Katzelnick
Shin-Ichiro Kawazu
Rudovick Kazwala
Thérèse Kearns
Karen Keddy
Jeremy Keenan
Paul Keim
Esther Kellenberger
Joshua Keller
Charles Kelly
Daryl Kelly
Terra Kelly
Louise Kelly-Hope
Alan Kemp
Volkhard Kempf
Malcolm Kennedy
Joanie Kenney
Ernest Kenu
Stella Kepha
Peter Kern
Natkunam Ketheesan
Abdelouahed Khalil
Mohammad Khalili
Ali Khamesipour
Asif Khan
Ricardo Khouri
Rudo Kieft
Margaret Kielian
Gerry Killeen
Dal Young Kim

Peter Kima
Akinori Kimura
Charles King
Christopher King
Jonas King
Jonathan King
Douglas Kinghorn
Luke Kingry
Amy Kirby
Louis Kirchhoff
Martyn Kirk
Theo Kirkland
Thomas Klei
Robyn Klein
Paul Klenerman
William Klimstra
Dan Kline
Michele Klingbeil
Amy Klion
Stephen Klotz
Darryn Knobel
Marcelo Knoff
Bart Knols
Barbara Knust
Wen-Chien Ko
Gary Kobinger
Julia Koehler
Cristian Koepfli
Alain Kohl
Nobuo Koizumi
Anne-Brit Kolsto
Nicholas Komar
Yoon Kong
Dimitrios Kontoyiannis
Bart Kooi
Kimberly Koporc
Steven Kopp
Daniel Korevaar
Pasi Korhonen
Titia Kortbeek
Jeroen Kortekaas
Andrew Kotze
Kouadio Kouame
Artemis Koukounari
Uriel Koziol
Moritz Kraemer
Peter Kraiczy
Laura Kramer
Peter Krause
Natacha Kremer
Alison Krentel
Manoj Krishnan
Alejandro Krolewiecki
Michael Kron
Jürgen Krücken
Adam Kucharski
Irene Kuepfer
Annette Kuesel
Katrin Kuhls
Jens Kuhn
Richard Kuhn
Senanayake Kularatne
Ambuj Kumar
Rajiv Kumar
Ravindra Kumar
Shailesh Kumar
Chi-Chien Kuo
Jonathan Kurtis
Ivan Kuzmin
June Kwon Chung
Mirjam Laager
A. Desiree Labeaud
Marcelo Labruna
Marcus Lacerda
Douglas Lacount
James Lacourse
Monique Lafon
Eric Lafontaine
William Lafuse
Jean-Christophe Lagier
Jonathan Lai
Aparna Lal
Marco Lalle
E. Laloy
Phung Lam
Xavier Lamballerie
Saba Lambert
Louis Lambrechts
Leah Lande
Scott Landfear
Zach Landis-Lewis
Jean Lang
Daniel Lang'o
Alexander Lankowski
Joseli Lannes-Vieira
Marion Lanteri
Olivier Lantz
Carlos Lanusse
Humberto Lanz-Mendoza
Maximilian Larena
Renee Larocque
Sébastien Larréché

Edmundo Larrieu
Estrella Lasry-Levy
Joerg Latus
Colleen Lau
Collen Lau
Francisco Laurindo
Catherine Lavazec
Anne Lavergne
Antti Lavikainen
Phillip Lawyer
Laura Layland
Helen Lazear
Maïna L'Azou
Claudio Lazzari
Thuy Le
Arnaud Le Menach
Epke Le Rutte
Monique Léchenne
Marc Lecuit
Bruce Lee
Han Lee
Jennifer Lee
John Lee
Keun Hwa Lee
Rogan Lee
Mariana Leguia
Veerle Lejon
Nadine Lemaitre
Jeffrey Lennon
Guillermo Leon
Christopher Lepczyk
Ganjana Lertmemongkolchai
Andres Lescano
Emilio Letang
William Letson
Bruno Levecke
Paul Levett
Adam Levine
Myron Levine
Rebecca Levine
Michael Levy
Bryan Lewis
Michael Lewis
Javier Lezaun
Pan Li
Joseph Liao
Daniel Libraty
Kelly Liebman
Marshal Lightowlers
Walter Lilenbaum
Siew Lim
Direk Limmathurosakul
Xiaoxu Lin
Johanna Lindahl
Brett Lindenbach
Steven Lindsay
Jacqueline Linnes
Bo Liu
Ching-Chuan Liu
Qin Liu
Qiyong Liu
Alejandro Llanos
Alejandro Llanos-Cuentas
Martin Llewellyn
Linda Lloyd
Leslie Lobel
Anne Lockyer
Melissa Lodoen
James Logan
P'Ng Loke
Eric Lombardini
Fabrizio Lombardo
Silvia Longhi
Jennifer Lord
J. Lorenzo-Morales
Jacob Lorenzo-Morales
Thomas Löscher
Alex Loukas
Leon P. Lounibos
Philip Loverde
Jenny Low
Nicola Low
Carl Lowenberger
Matthew Lozier
Jiahai Lu
Alison Luce-Fedrow
Juan Ludert
Helena Lugão
Julius Lukes
Lucy Lum
Nongkran Lumjuan
Zhao-Rong Lun
Britta Lundström-Stadelmann
Joel Lutomiah
Manfred Lutz
Shari Lydy
David C. Lye
David Mabey
Kevin Macaluso
Fabiana Machado
Carlos Machain-Williams
Fernando Macian

Ian Mackay
Charles Mackenzie
Stephen Mackessy
Erich Mackow
Colin Macleod
Alhaji Madara
Louis Maes
Margaret Mafe
Stephen Magesa
Stefan Magez
Ulf Magnusson
Siddhartha Mahanty
Sean Maher
Carla Maia
Robbie Mailliard
Rick Maizels
Laleh Majlessi
Joanne Maki
Gathsaurie Malavige
Rosa Maldonado
Indu Malhotra
Benoit Malleret
David Malone
Robert Malone
Yukari Manabe
Mark Manary
Lenore Manderson
Brian Mann
Jennifer Manne
Carrie Manore
Pablo Manrique Saide
Pablo Manrique-Saide
Karen Mansfield
Sunny Mante
Jean-Claude Manuguerra
Liang Mao
Duncan Mara
Madhav Marathe
Alessandro Marcello
Paula Marcet
Jonathan Marchant
Antonio Marco
Elaine Marcos
Eloi Marijon
Wilfred Marissen
Lewis Markoff
Wanda Markotter
Florian Marks
Michael Marks
Christina Marra
Thomas Marrie
Robyn Marsh
Vito Martella
Celina Martelli
Matthias Marti
Coralie Martin
Diana Martin
Julio Martin
Richard Martin
Eric Martinez
Maria Del Mar Martínez Y García
I. Martinez-Pino
Jaime Martinez-Urtaza
Inés Martín-Martín
Francisco Rogerlândio Martins-Melo
Santiago Mas-Coma
Collen Masimirembwa
Dmitri Maslov
Fekadu Massebo
Pietro Mastroeni
Anuja Mathew
Derrick Mathias
Vitor Mati
Greg Matlashewski
Laura Matrajt
Mariko Matsui
Keith Matthews
Michael Matthias
Richard Maude
Ian Maudlin
Isabel Mauricio
Radheshyam Maurya
Stephanie Mauti
Michael Maze
Pamela Mbabazi
Evaristus Mbanefo
Deborah Mc Farland
John McBride
Laura-Isobel McCall
Christina McCarthy
Geoff McCaughan
Elizabeth McClure
Glenn McConkey
Malcolm McConville
Judith McCool
Michael McCracken
John McCreadie
Emily McDonald
Lorraine McElhinney
Jimmy McGuire
Bradford McGwire
Heather McKay

Don McManus
W. Robert McMaster
Conor McMeniman
Tom McNeilly
Kirsty McPherson
Henry McSorley
Stephen McSorley
Paul McVeigh
Robert Meagher
Oleg Mediannikov
Graham Medley
James Meegan
Heinz Mehlhorn
Rojelio Mejia
Marcia Melhem
Breno Mello
Clelia Mello-Silva
Martin Meltzer
Ella Mendelson
Ian Mendenhall
Rinaldo Mendes
Tiago Mendes
Fela Mendlovic
Gustavo Menezes
Daniel Menezes-Souza
Habtamutaddele Menghistu
Joris Menten
Corinne Mercier
Valeria Meroni
Pascal Mertens
François-Xavier Meslin
Louisa Messenger
Lynne Messer
Jane Messina
Raphaelle Metras
Virginie Michel
Nicholas Midzi
Luis Mier-y-Teran
Stephanie Migchelsen
Andrei Mihalca
Sebastian Mikolajczak
Matthieu Million
James Mills
Michael Minnick
Chad Mire
Mehdi Mirsaeidi
Usha Misra
Nerges Mistry
Kate Mitchell
Oriol Mitjà
Edward Mitre
Maurice Mittlemark
Harran Mkocha
Farrokh Modabber
Don Moerman
Vittal Mogasale
Mamoun Mohamed Ali Homeida
Hamish Mohammed
Alok Mohapatra
Igor Mokrousov
Heidrun Moll
Dinesh Mondal
Adriano Mondini
Juthathip Mongkolsapaya
Carlos Montanari
Fernando Monteiro
Wuelton Monteiro
Susan Montgomery
Lucia Montoya
Antonio Montresor
Robert W. Moon
Cindy Moore
Sean Moore
Susan Moore
Ann Moormann
Eddy Moors
Siobhan Mor
Milton Moraes
Paula Moraga
Miguel Morales
Serge Morand
Alessandra Morassutti
Ricardo Moratelli
Dana Mordue
Luciano Moreira
Otacilio Moreira
Javier Moreno
Max Moreno Madriñán
David Morens
Katy Morgan
Lisa Morici
Cory Morin
Pedro Moro
Susan Morpeth
John Morrey
J. Glenn Morris Jr.
Amy Morrison
Brian Morrison
Thomas Morrison
Lydia Mosi
David Mosser
Vladimir Motin

Jeremy Mottram
Adrian Mountford
Charles Mowbray
Andrés Moya
Caleb Mpyet
Atis Muehlenbachs
Ivo Mueller
Thomas Mueller
Claire Mugasa
Julius Mugwagwa
Dennis Muhanguzi
Samson Mukaratirwa
Rita Mukhopadhyay
Suchetana Mukhopadhyay
Grace Mulcahy
Albert Mulenga
Joachim Müller
Kristin Mullins
Dieudonné Mumba
Beatriz Munoz
Jose Munoz
Jorge Munoz-Jordan
Vincent Munster
Leonard Munstermann
T. Murali
C. Murdock
Antonio Muro
Edward Murphy
Gerald L. Murray
Silvane Murta
Ahmed Musa
Anwar Musah
Carlos Muskus
Didier Musso
Karuppiah Muthumani
Amos Deogratius Mwaka
Victor Mwanakasale
Peter Myler
Ben Naafs
Mathieu Nacher
Susan Nadin-Davis
Jyothi Nagajyothi
Rana Nagarkatti
Hristo Najdenski
Minoru Nakao
Noboru Nakasone
Yoshinori Nakazawa
Hira Nakhasi
Jarlath Nally
Subhadra Nandakumar
Miya Narushima
Scott Nash
Stephen Nash
Theodore Nash
Indira Nath
Sheila Nathan
Ruvandhi Nathavitharana
Elena Naumova
Miguel Navarro
Momar Ndao
Martial Ndeffo Mbah
Daniel Neafsey
Obiageli Nebe
Deborah Negrão-Corrêa
P. Nejsum
Kenrad Nelson
Nicole Nemeth
Vivek Nerurkar
Malden Nesheim
Heinrich Neubauer
Paul Newton
Johan Neyts
Lee-Ching Ng
Lisa Ng
Patrick Nguku
Pin Nie
Matthias Niedrig
Birgit Nikolay
Alasdair Nisbet
Alfred K. Njamnshi
M. Njenga
Doris Njomo
Joo Hwan No
James Noah
Aline Nobre
Harold Noel
Maurício Nogueira
María Mercedes Nogueras
Matthew Nolan
Justin Nono
Abdisalan Noor
Rahmah Noordin
Phillip Norris
Robert Norton
Antoine Nougairède
Pierre Nouvellet
Mairi Noverr
Richard Novick
Oscar Noya
Harry Noyes
Tomoyoshi Nozaki
Jack Nunberg

Márcio Nunes
Patricia Nuttall
Davis Nwakanma
Bertram Ekejiuba Bright Nwoke
Hellen Nyakundi
Melissa Nyendak
Susanne Nylen
Daniel O'Brien
David O'Callaghan
Clara Ocampo
Elise O'Connell
Peter Odermatt
Stephanie Ogden
Olumide Ogundahunsi
Nobuo Ohta
Kazunori Oishi
Joseph Okeibunor
Iruka Okeke
Kendi Okuda
Gaetano Oliva
Kevin Olival
Guilherme Oliveira
Maria Oliveira
Alicia Olivier
Martin Olivier
Stephan Ölschläger
David Olson
Donald Olson
Sandra O'Neill
Eng Eong Ooi
Maurice Ope
Ian Orme
Yoshio Osada
Kara Osbak
Graciela Ostera
Bart Ostyn
William Oswald
Jose Oteo
Domenico Otranto
Eric Ottesen
Marc Ouellette
Johnson Ouma
Anna Overby
Hans Overgaard
Ukam Oyene
Yusuf Ozbel
Christopher Paddock
Slobodan Paessler
Daniela Pagnozzi
Hélène Pailhoriès
Subhamoy Pal
Utpal Pal
Ricardo Palacios
Clarisa Palatnik-De-Sousa
Andres Palencia
Mark Pallen
Abhishek Pandey
John Panepinto
Bo Pang
Junxiong Pang
S.P. Pani
Jyoti Panta
Eric Pante
Philippos Papathanos
Claudia Paredes Esquivel
David Parenti
Daniel Paris
Andrew Park
Om Parkash
Tom Parks
Christopher Parry
Marilyn Parsons
Shama Parveen
Parviz Parvizi
David Pascual
Mercedes Pascual
Luiz Passero
Macej Pastuszczak
Jaymin Patel
Beverly Paterson
Steve Paterson
Arunasalam Pathmeswaran
Pabitra H. Patra
Pradeep Patra
Kimberly Paul
Richard Paul
Christophe Paupy
Janusz Paweska
Valerie Paz-Soldan
Lori Peacock
Mark Pearson
Jorge Pedrosa
Rosanna Peeling
Maria Peichoto
Henry Peixoto
Pablo Peixoto
Jose Pelegrino
Julien Pelletier
Krisana Pengsaa
Maria Lucia Penna
Pamela Pennington
Marcos Pereira

Ricardo Pereira
Rushika Perera
Ana Pérez
José Pérez-Victoria
Alex Perkins
Felipe Pessoa
Nathan Peters
Ruth Peters
Brett Petersen
Townsend Peterson
Marie-Agnès Petit
Linda Petrone
Michael Pfaller
Kenneth Pfarr
Isaac Phiri
Renaud Piarroux
Roxane Piazza
Brett Pickett
Sacha Pidot
Raymond Pierce
Otávio Pieri
Ted Pierson
Theodore Pierson
David Pigott
Dulce Pimentel
Clemencia Pinilla
Somchai Pinlaor
Benjamin Pinsky
Sebastien Pion
R. Pitts
Virginia Pitzer
Alexander Pletnev
Piero Poletti
Rafael Polidoro
Christelle Pomares
Alicia Ponte Sucre
Travis Porco
Françoise Portaels
Kevin Porter
Damien Portevin
Erik Post
Miriam Postan
Jeffrey Powell
Ann Powers
Doriraj Prabhakaran
Vijay Prajapati
Kashi Prasad
Kashi N. Prasad
Siddharth Pratap
Gabriele Prati
Clarissa Prazeres Da Costa
Elisa Prestes
Pierre-Marie Preux
Erin Price
Ric Price
David Pride
Jeffrey Priest
Todd Primm
Gerardo Priotto
Supannee Promthet
Anna Protasio
Natalie Prow
Miguel Prudencio
Andrea Pugliese
Bali Pulendran
Geoffrey Puzon
Alyssa Pyke
Firdausi Qadri
Men-Bao Qian
Juarez Antonio Quaresma
Tom Quinn
Rupert Quinnell
Juan Carlos Quintana Castillo
Najma Rachidi
Justin Radolf
Senaka Rajapakse
Radha Rajasingham
Vedantam Rajshekhar
Kapa Ramaiah
Srinivasan Ramakrishnan
José Luis Ramirez
Juan Ramirez
Juan David Ramirez
Marcel Ramirez
Celso Ramos
Isabela Ramos
Claudia Rangel-Escareño
Hilary Ranson
Ramakrishna Rao
Chad Rappleye
Raffaella Ravinetto
Julian Rayner
Hugo Razuri
Fabio Re
Jennifer Read
Jonathan Read
Laurie K. Read
Paul Ready
Fernando Real
Stanislas Rebaudet
Maria Rebollo Polo
Sergio Recuenco

Steven Reed
Erin Rees
Tiffany Reese
Lisa Reimer
Neil Reiner
Robert Reiner
Alexandre Reis
William Reisen
Michael Reiskind
Paul Reiter
Klaus Reither
Barth Reller
Franck Remoue
Silvia Repetto
Jose Requena
Serge Resnikoff
Angela Restrepo
Giovanni Rezza
Antonio Ribeiro
Isabela Ribeiro
Carlos André Ornelas Ricart
Natasha Ricci
Guerrant Richard
Allen L. Richards
Frank Richards
Jack Richards
S. Richards
Rebecca Richards-Kortum
Rebecca Rico-Hesse
Michael Riehle
Basista Rijal
Gabriel Rinaldi
David Rinker
Sébastien Rioux Paquette
Scott Ritchie
Koert Ritmeijer
Manuel Ritter
Manuel Rivas
Carlos Robello
Michael Robert
Leanne Robinson
Mark Robinson
Justin Roby
Kat Rock
Daniel Rockey
Barry Rockx
Isabel Roditi
Anderson Rodrigues
Fernando Rodrigues
Nilton Rodrigues
Ana Rodriguez
Juan Rodriguez
Isabel Rodriguez-Barraquer
Mario Rodríguez-Lopez
Mario Rodriguez-Perez
Silvia Rodríguez-Ramilo
Dawn Roellig
Meta Roestenberg
Michael Rogan
Matthew Rogers
Christophe Rogier
Iva Rohousova
Pierre Rollin
David Rollinson
Lucia Romani
Camila Romano
Patricia Romano
Gustavo Romero
Anne Rompalo
Catherine Ronet
Pornpimol Rongnoparut
Kerry Rood
Bruce Rosa
Patricia Rosa
Rebecca Rose
Mara Cecilia Rosenzvit
Allen Ross
Gabriele Rossi
Omar Rossi
Shannan Rossi
Alexa Rosypal
Martina Rothenbühler
Dominique Rousset
Francoise Routier
Syamal Roy
Akiiki Rubaire
Giovina Ruberti
Eric Rubin
Jai Rudra
Andrea Ruecker
Marie-Thérèse Ruf
José Antonio Ruiz-Postigo
Nick Ruktanonchai
Silvia Runge-Ranzinger
Philip Russell
Tanya Russell
Graciela Russomando
Steven Rutherford
Peter Ryan
Una Ryan
Kristyna Rysava
Vagner Sá

Elmar Saathoff
Karla Saavedra-Rodriguez
Claude Sabeta
David Sack
Moussa Sacko
David Sacks
Stephen Safe
Masayuki Saijo
Talia Sainz
Yasuhito Sako
Krzysztof Sakrejda
Anu Sakthianandeswaren
Naseem Salahuddin
Rehana Salam
Bashir Salim
Henrik Salje
Jeanne Salje
Gabriela Salmon-Mulanovich
Roberto Salvatella
Vittorio Sambri
Richard Samuels
John Samuelson
Ana Sanchez
Elda Sanchez
Cecilia Sánchez
Nuria Sanchez Clemente
Suzanne Sandmeyer
Saikolappan Sankaralingam
Mauricio Sant'Anna
Vamilton Alvares Santarém
Gilberto Santiago
Helton Santiago
Maria Soledad Santini
Saul Santivanez
Marcelo Santoro
Flavia Santos
Fred Santos
Renato Santos
Valeria Saraceni
Vanessa Sarathy
Nancy Saravia
Fred Sarfo
Carlos Sariol
Rajiv Sarkar
Euzenir Sarno
Derek Sarovich
Smitha Sasindran
Yorifumi Satou
Benjamin Satterfield
Silvia Sauleda
Paul Saunderson
Kittisak Sawanyawisuth
Somphou Sayasone
Ilana Schafer
Francis Schaffner
Huub Schellekens
Esther Schelling
Sergio Schenkman
Manuel Schibler
John Schieffelin
Alejandro Schijman
Stefan Schild
Connie Schmaljohn
Ralf Schmid
Nathan Schmidt
Matthias Schnell
Randal Schoepp
Frank Scholle
Tony Schountz
Vern Schramm
Albert Schriefer
Simone Schuller
Claudia Schulz
Verena Schünemann
Tom Schwan
David Schwartz
Eli Schwartz
Ira Schwartz
Edda Sciutto
Pamela Scorza
Alan Scott
Janet Scott
Thomas Scott
Gavin Screaton
James Scriven
Kimberley Seed
Karin Seifert
Richard Selby
Angamuthu Selvapandiyan
Saul Semiao-Santos
Roshanak Semnani
Denis Sereno
Silatham Sermrittirong
David Serre
Alessandro Sette
Anaiá Sevá
Karl Seydel
Seyyed Javad Seyyedtabaei
Mara Shainheit
Sifat Sharmin
Tyler Sharp
Jeffrey Shaw

Thomas Shelite
Jilong Shen
Donald Shepard
Pei-Yong Shi
Mineko Shibayama
Brian Shiels
Clive Shiff
Arti Shinde
Sadegh Shirian
Gabriel Shirima
Ziiv Shkedy
Sourya Shrestha
Pei-Yun Shu
Kathryn Shuford
Stephanie Shwiff
Elisa Sicuri
Issa Sidibe
Constantinos Siettos
Aita Signorell
Mar Siles-Lucas
Ana Clara Silva
Claudia Lucia Silva
Shuzhen Sim
Rachel Simmonds
Cameron Simmons
Graham Simmons
Gustave Simo
Augusto Simoes-Barbosa
Fabrice Simon
Raphael Simon
Calvin Sindato
Merrill Singer
Rachael Singer
Steven Singer
Brajendra Singh
Gagandeep Singh
Neeloo Singh
Pushpendra Singh
Rakesh Singh
Ruchi Singh
Sarman Singh
Steven Sinkins
Vitali Sintchenko
André Siqueira
Herbert Siqueira
Jair Siqueira-Neto
Chukiat Sirivichayakul
Paiboon Sithithaworn
Patrick Skelly
Marvin Sklar
Barton Slatko
Jonathan Sleeman
Tom Slezak
Mark Slifka
C. Smales
Pamela Small
Timo Smieszek
Duncan Smith
Judith Smith
Peter Smith
Peter Smooker
Michael Smout
Georges Snounou
Rodrigo Soares
Cristina Socolovschi-Audoly
Laia Solano-Gallego
Aldo Solari
Hernán Solari
Anthony Solomon
Johannes Sommerfeld
Daniel Sonenshine
Haihan Song
Lynn Soong
Ghislain Sopoh
Carlos Sorgi
Teshael Sori
Javier Sotillo
Marc Souris
Danielle Souza
Leonardo Souza
Reinaldo Souza-Santos
Robert Spear
John Spencer
Jessica Spengler
Niko Speybroeck
Markus Spiliotis
David Spray
Armand Sprecher
Anon Srikiatkhachorn
Simona Stäger
Jason Stajich
Lola Stamm
Miles Stanford
Michelle Stanton
Anette Stauch
Andrew Steer
Marina Stein
Mark Stenglein
Christopher Stephens
Jeremy Sternberg
Sharon Stevelink
Kim Stevens

Lori Stevens
Brian Stevenson
Aisha Stewart
Ymkje Stienstra
Jonathan Stiles
Wilma Stolk
Craig Stoops
Nadia Storm
J. Russell Stothard
Mary Straub
Daniel Streblow
Gillian Stresman
Dan Strickman
Clare Strode
Jim Strong
Nathalie Strub-Wourgaft
Claudio Struchiner
Hugh Sturrock
Chunlei Su
V. Nathan Subramaniam
Philip Suffys
Andreas Suhrbier
Nancy Sullivan
David Sullivan Jr.
Robert Sumaye
Gui-Quan Sun
Peifang Sun
Appavu Sundaram
Cord Sunderkoetter
Sujai Suneetha
Pra-on Supradish
Dipika Sur
Mehul Suthar
Claudette Sutherland
Yasuhiro Suzuki
Staffan Svard
Subramanian Swaminathan
Andrea Swei
Zainulabeuddin Syed
Walter Tabachnickk
Earnest Tabah
Hiroshi Tachibana
Fabienne Tachini-Cottier
Wanderli Tadei
Fabien Taieb
Ratree Takhampunya
Kawsar Talaat
Benoit Talbot
Francesca Tamarozzi
Ernest Tambo
Cheong Huat Tan
Choo Hock Tan
Gladys Tan
Yi Tan
Sirikachorn Tangkawattana
Herbert Tanowitz
Amilcar Tanuri
Rick Tarleton
Utpal Tatu
Iman Tavassoly
Bineyam Taye
Hugh Taylor
Jesse Taylor
Katherine Taylor
Matthew Taylor
David Taylor-Robinson
Weldemelak Tedla
Antonio Teixeira
Maria Glória Teixeira
Santuza Teixeira
Andréa Teixeira-Carvalho
Sam Telford III
Erich Telleria
Quirine Ten Bosch
Olle Terenius
Luis Terrazas
Kevin Tetteh
Larissa Thackray
Janjira Thaipadungpanit
Stig Milan Thamsborg
Saravanan Thangamani
Oriel Thekisoe
Steven Theriault
Lian Thomas
Saijo Thomas
Stephen Thomas
John Thomas III
Peter Thompson
Richard Christopher Thompson
Emma Thomson
Russell Thomson
Panpim Thongsripong
Hans-Hermann Thulke
Huaiyu Tian
Paul Timmings
Matthew Todd
Kentaro Tohma
Ana Tomás
Kay Tomashek
Nicolás Tomasini
Stephen Tompkins
Abebayehu Tora

Noel Tordo
Paul Torgerson
Katherine Torres
José Tort
Baldwyn Torto
Pablo Tortosa
Yesim Tozan
Rebecca Traub
Luiz Travassos
Donato Traversa
Bruno Travi
Vianney Tricou
Lucienne Tritten
Adriana Troyo
Konstantin Tsetsarkin
Takafumi Tsuboi
Moriya Tsuji
Zhijian Tu
Apichai Tuanyok
Joseph Tucker
Ye Tun
Hugo Turner
Joseph Turner
Florencio Ubeira
Uade Ugbomoiko
Silvia Uliana
Buddy Ullman
Rainer Ulrich
Rich Umeh
Eduardo Undurraga
Thomas Unnasch
Priya Uppuluri
Joseph Urban
Julio Urbina
Jürg Utzinger
Tim Uyeki
Nestor Uzcategui
Tushar Vaidya
Marilene Vainstein
Glyn Vale
Jesus Valenzuela
Isabelle Vallee
Gustavo Vallejo
Steven Valles
Rajesh Valmiki
Olaf Valverde Mordt
Wim Van Bortel
Wim van Brakel
Wendy van de Sande
Andrew van den Hurk
Gert Van der Auwera
Wim van der Poel
Tjip van der Werf
Johan van Griensven
Lisette van Lieshout
Philippe Van Lint
Pieter-Paul van Thiel
Wesley Van Voorhis
Johan Van Weyenbergh
Katrien Vanbocxlaer
Kalwaje Eshwara Vandana
Koen Vandelannoote
Thomas Vanderford
Alain Vanderplasschen
David Vandroux
Nongnuch Vanittanakom
Dana Vanlandingham
Sophie Vanwambeke
Elizabeth VanWormer
Ravi Vasanthapuram
Dewton Vasconcelos
Nikos Vasilakis
Subhash Vasudevan
Jefferson Vaughan
Meagan Vaughn
Muriel Vayssier-Taussat
Susana Vaz Nery
Gonzalo Vazquez-Prokopec
Angela Veesenmeyer
Aristea Velegraki
Yael Velleman
Sandhya Venkatswami
Jonathan Vennerstrom
Maria Teresa Ventura
Lucio Vera-Cabrera
Patricia Veras
Kristien Verdonck
Sergio Verjovski-Almeida
Rajesh Verma
Sebastian Vernal
Kenneth Vernick
Guilherme Verocai
Jaco Verweij
Agostinho Viana
Mafalda Viana
Diego Viasus
Cecile Viboud
Aurelien Vigneron
Paluru Vijayachari
Stalin Vilcarromero
Sharon Yvette Angelina Villanueva
Luis Eduardo Martinez Villegas

Joseph Vinetz
Mark Viney
Rodolfo Viotti
Marcus Virmond
Natalia Voge
Petr Volf
Veronika Von Messling
Lorenz Von Seidlein
Sirenda Vong
John Vontas
Neil Vora
H. Martin Vordermeier
Jan Votypka
Liina Voutilainen
Dominique Vuitton
Ajai Vyas
Jesse Waggoner
David Wagner
John Waldeisen
Etienne Waleckx
Anthony Walker
David Walker
Edward Walker
Martin Walker
Thomas Walker
Ryan Wallace
Lance Waller
Judd Walson
Katharine Walter
William Walton
Yvonne Walz
Welcome Wami
Chengming Wang
Qian Wang
Robert Wang
Shue Wang
Wei-Kung Wang
Yanhai Wang
Mark Wansbrough-Jones
Jonathan Warawa
Honorine Ward
Jordan Ward
Michael Ward
David Warhurst
David Warrilow
Marion Wassermann
Toshiki Watanabe
Steve Waterman
Ray Waters
David Watkins
Alice Wattam
Douglas Watts
Matthew Watts
Helen Wearing
Jill Weatherhead
Cameron Webb
Friedemann Weber
Bonnie Webster
Gareth Weedall
David Weetman
Tiffany Weinkopff
Daniela Weiskopf
Brian Weiss
Matthew Weitzman
Melanie Wellington
Chad Wells
Oliverio Welsh
Hao Wen
Shih-Feng Weng
Karl Werbovetz
Guilherme Werneck
Catherine Werts
Dawn Wesson
Dennis West
Sheila West
Judith White
Laura White
Nicholas White
James Whitney
Douglas Widman
Giovanni Widmer
W. Wiersinga
Stephen Wikel
Annelies Wilder-Smith
Patricia Wilkins
Craig Williams
David Williams
Diana Williams
Kim Williamson
Heather Williamson-Jordan
Arve Willingham
Rodney Willoughby
Bridget Wills
Mary Wilson
Shona Wilson
Jennifer Wilson-Welder
Cheryl Winkler
Mirko Winkler
Peter Winskill
Jeffrey Withey
Michael Witty
Dawit Wolday

Kimberly Won
Charles Wondji
Kum Thong Wong
Sirichit Wongkamchai
James Wood
Christopher Woods
R. Wooten
Gary Wormser
Henry Wortis
Brendan Wren
Christine Wright
Dr. Xianfu Wu
Susan Wyllie
Zhiyong Xi
Junchao Xia
Cecilia Ximenez
Cecilia Ximénez
Xiaolu Xiong
Zhiheng Xu
Feng Xue
Bala Yabo
Sophie Yacoub
Zaida Yadón
Laith Yakob
Hiroshi Yamasaki
Chih-Wei Yang
Kun Yang
Wan Yang
Zhicong Yang
S. Yanow
Cedric Yansouni
Chaoqun Yao
Peiling Yap
Richard Yapi
Phillip Yates
Dorothy Yeboah-Manu
Janet Yee
Emmanuel Yenshu
Mitsutoshi Yoneyama
In-Kyu Yoon
Nobuko Yoshida
Timothy Yoshino
Neil Young
Paul Young
Xue-Jie Yu
Jing Yuan
Thomas Yuill
Sherif Zaki
Martha Zakrzewski
Keivan Zandi
Qian Zhang
Yaobi Zhang
Elyes Zhioua
Guangming Zhong
Hua Zhong
Yanjiao Zhou
Guan Zhu
Emily Zielinski-Gutierrez
Eduard Zijlstra
Dan Zilberstein
Jacques Zimmer
Peter Zimmerman
Bianca Zingales
Russolina Zingali
Kate Zinszer
Carina Zittra
Simona Zompi
Carmen Zorrilla
